# Interpretable machine learning method to predict the risk of pre-diabetes using a national-wide cross-sectional data: evidence from CHNS

**DOI:** 10.1186/s12889-025-22419-7

**Published:** 2025-03-26

**Authors:** Xiaolong Li, Fan Ding, Lu Zhang, Shi Zhao, Zengyun Hu, Zhanbing Ma, Feng Li, Yuhong Zhang, Yi Zhao, Yu Zhao

**Affiliations:** 1 School of Public Health, Ningxia Medical University, Yinchuan Ningxia, 750004 China; 2 NHC Key Laboratory of Metabolic Cardiovascular Diseases Research, Ningxia Medical University, Yinchuan, 750004 China; 3 Ningxia Key Laboratory of Environmental Factors and Chronic Disease Control, Yinchuan Ningxia, 750004 China; 4 School of Public Health, Tianjin Medical University, Tianjin, 300070 China; 5 School of Public Health, Shanghai Jiao Tong University, Shanghai, 200025 China; 6 School of Basic Medicine, Ningxia Medical University, Yinchuan Ningxia, 750004 China; 7 Department of Laboratory Medicine, General Hospital of Ningxia Medical University, Yinchuan Ningxia, 750004 China

**Keywords:** Pre-diabetes, LASSO regression, XGBoost model, Prediction, Shapley additive explanation

## Abstract

**Objective:**

The incidence of Type 2 Diabetes Mellitus (T2DM) continues to rise steadily, significantly impacting human health. Early prediction of pre-diabetic risks has emerged as a crucial public health concern in recent years. Machine learning methods have proven effective in enhancing prediction accuracy. However, existing approaches may lack interpretability regarding underlying mechanisms. Therefore, we aim to employ an interpretable machine learning approach utilizing nationwide cross-sectional data to predict pre-diabetic risk and quantify the impact of potential risks.

**Methods:**

The LASSO regression algorithm was used to conduct feature selection from 30 factors, ultimately identifying nine non-zero coefficient features associated with pre-diabetes, including age, TG, TC, BMI, Apolipoprotein B, TP, leukocyte count, HDL-C, and hypertension. Various machine learning algorithms, including Extreme Gradient Boosting (XGBoost), Random Forest (RF), Support Vector Machine (SVM), Naive Bayes (NB), Artificial Neural Networks (ANNs), Decision Trees (DT), and Logistic Regression (LR), were employed to compare predictive performance. Employing an interpretable machine learning approach, we aimed to enhance the accuracy of pre-diabetes risk prediction and quantify the impact and significance of potential risks on pre-diabetes.

**Results:**

From the China Health and Nutrition Survey (CHNS) data, a cohort of 8,277 individuals was selected, exhibiting a disease prevalence of 7.13%. The XGBoost model demonstrated superior performance with an AUC value of 0.939, surpassing RF, SVM, DT, ANNs, Naive Bayes, and LR models. Additionally, Shapley Additive Explanation (SHAP) analysis indicated that age, BMI, TC, ApoB, TG, hypertension, TP, HDL-C, and WBC may serve as risk factors for pre-diabetes.

**Conclusion:**

The constructed model comprises nine easily accessible predictive factors, which prove highly effective in forecasting the risk of pre-diabetes. Concurrently, we have quantified the specific impact of each predictive factor on the risk and ranked them based on their influence. This result may serve as a convenient tool for early identification of individuals at high risk of pre-diabetes, providing effective guidance for preventing the progression of pre-diabetes to T2DM.

**Supplementary Information:**

The online version contains supplementary material available at 10.1186/s12889-025-22419-7.

## Introduction

In recent decades, the incidence and mortality rates of diabetes have been steadily increasing globally, attributed to shifts in lifestyle and dietary habits. Consequently, diabetes has emerged as a pervasive public health concern worldwide. According to the International Diabetes Federation (IDF), the global number of people with diabetes reached 451 million in 2017 and will rise to 693 million in 2045 [1]. China has the largest number of diabetic patients in the world. In the past 10 years (2011–2021), the number of diabetic patients in China has increased from 90 million to 140 million, an increase of 56%, of which about 72.83 million patients have not yet been diagnosed, a proportion of 51.7% [2]. It is anticipated that over the next 20 years, the prevalence of diabetes in China will decline, but by the year 2030, it is projected to reach 164 million, and by 2045, it is expected to reach 175 million [2]. This trend has garnered public attention, positioning diabetes as the third most common non-communicable disease [3]. According to the “International Diabetes Federation Global Diabetes Map (10th Edition) 2021”, China incurs an annual direct medical expenditure of up to 165.3 billion US dollars related to diabetes [2], with a staggering 95% allocated to treating complications. This imposes a significant social and economic burden. Public health interventions for diabetes prevention represent the optimal approach for early intervention [4–6]. Increasing evidence suggests that lifestyle modifications can prevent or delay the prevalence of Type 2 Diabetes Mellitus (T2DM) [7]. The transition from early metabolic abnormalities, impaired fasting glucose (IFG), and impaired glucose tolerance (IGT), to diabetes may take several years [8]. Pre-diabetes, characterized by IFG, is one of the most critical high-risk groups for diabetes, with an annual transition rate of 1.5–10% from IFG to T2DM. Therefore, early screening, prediction, and intervention for potential pre-diabetic individuals in the general population can improve the quality of life and alleviate the economic burden of T2DM and its complications.

Timely identification of high-risk individuals and targeted prevention efforts are beneficial for predicting the risk of pre-diabetes in clinical practice or community screening [9–11]. Therefore, pre-diabetes risk prediction models are widely used in healthcare to screen high-risk populations. These models help identify pre-diabetes patients in advance, allowing timely action to prevent or delay the onset of diabetes and its chronic complications. In recent years, with the wide application of data mining techniques in healthcare utilization, an increasing number of statistical models and machine learning algorithms have been applied to research on pre-diabetes risk prediction and identification of risk factors [12–14]. For example, Heikes et al. [15] investigated a tool for predicting diabetes risk using undiagnosed and pre-diabetes data in the United States. Birk N et al. employed several machine learning and statistical methods to calculate the Global Diet Quality Score (GDQS) and screen for pre-diabetes using FFQ survey data [16]. Numerous studies indicate that the risk factors for pre-diabetes mainly include unhealthy diet, age, family history of diabetes, race, obesity, sedentary lifestyle, and history of gestational diabetes [17–19]. Previous research has also reported the associations among sex, body mass index (BMI), pregnancy, and metabolic status with pre-diabetes [20, 21]. However, the existing pre-diabetes risk models are limited to known risk factors or traditional statistical methods, and those prediction studies were not precise enough and less interpretable.

Machine learning algorithms, such as Support Vector Machines (SVM), Naive Bayes (NB), Random Forest (RF), Artificial Neural Networks (ANNs), Decision Trees (DT), and XGBoost, have been widely employed in various complex data analytics tasks due to their excellent generalization and discriminative abilities in dealing with high-dimensional data. These algorithms can effectively capture and analyze the multidimensional features of patients’ health status, thus providing more accurate descriptions of the health status [22]. Zou et al. [23] used machine learning methods to predict diabetes in Luzhou, China and validated the model through five-fold cross-validation. Nguyen BP et al. [24] utilized deep learning algorithms to predict diabetes incidence, demonstrating that complex methods can improve model performance. Choi et al. developed two pre-diabetes screening models using artificial neural networks (ANNs) and support vector machines (SVM), systematically evaluating the models through internal and external validation [25]. De Silva et al. employed feature selection and machine learning methods to identify predictive factors for pre-diabetes in a nationally representative sample of the US population [26]. These studies have contributed to the development of advanced machine learning and deep learning models, which can predict diabetes more accurately than traditional methods. However, existing methods may lack explanations for the potential mechanisms of risk factors and may suffer from insufficient and low-quality data, thereby limiting the performance and applicability of predictive models. Therefore, it is essential to establish effective predictive models to identify risk factors for pre-diabetes and quantitatively explore the impact of interpretable factors. In addition, our research not only focuses on the predictive power of the models but also aims to improve the interpretability of the model so that healthcare professionals can understand and trust the model’s predictions. By employing advanced data processing techniques and multidimensional risk analysis, our model aims to be a powerful support for clinical decision-making and to guide future research directions to further explore and explain the complex risk factors of prediabetes.

Motivated by the above discussion, in this study, we aimed to employ six machine learning algorithms (RF, SVM, DT, Naive Bayes, ANNs, and XGBoost) to improve the accuracy of the risk prediction model of pre-diabetes and evaluated the performance of various algorithms through 5-fold cross-validation. Then we quantitatively assess the contribution of factors to pre-diabetes risk using the interpretable machine learning method SHAP. The analysis includes nine readily accessible predictive indicators, including biochemical and physical examination data. Five indicators (AUC, F1 score, accuracy, sensitivity, and specificity) were reported to compare the prediction performance. The SHAP method was used to quantify the impact and importance of pre-diabetes risk predictors. These results can aid in identifying high-risk pre-diabetes individuals in the general population and provide effective and operability early intervention measures for pre-diabetes.

## Objectives and methods

### Research objectives

**Fig. 1:**
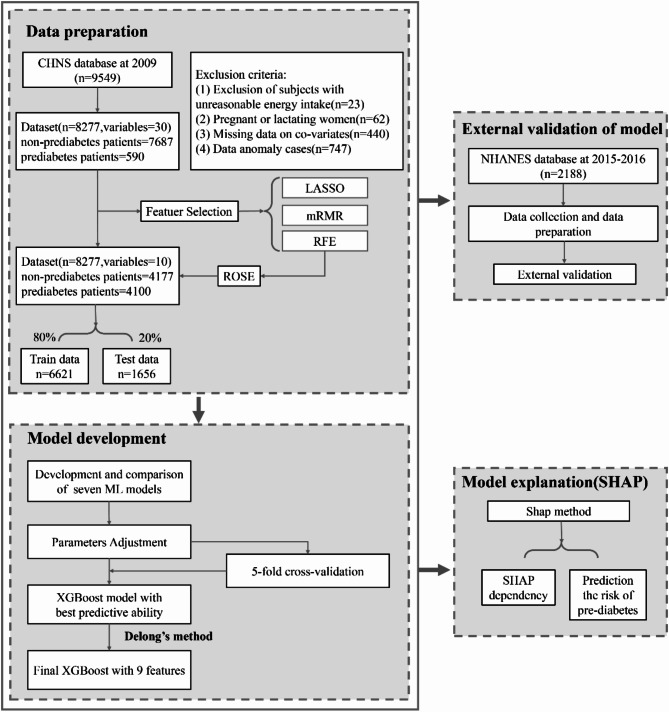
The process of data analysis based on an interpretable machine-learning method

The China Health and Nutrition Survey (CHNS) is a family-based, prospective study conducted in 1989, 1991, 1993, 1997, 2000, 2004, 2006, 2009, 2011, and 2015 [27]. The data were published on http://www.cpc.unc.edu/projects/china. In this study, 8,277 cases in the China Health and Nutrition Survey database from Jan. 1 to Dec. 31 2009 were selected as the research objects. The information of individual participants, which has been desensitized, could not be identified during or after data collection. Exclusion criteria: (1) Exclusion of subjects with unreasonable energy intake; (2) Pregnant or lactating women; (3) Missing data on co-variates; (4) Data anomaly cases (see S1 Fig for more details). Pre-diabetes was identified by fasting blood glucose ≥ 6.1 mmol/L, < 7.0 mmol/L [28], hypertension was diagnosed as average systolic BP/diastolic BP ≥ 140/90 mmHg [29], the process of the participant’s selection was shown in Fig. 1. The variable assignments and descriptions for the dataset are shown in the Table S1.

### Dataset partition

**Fig. 2:**
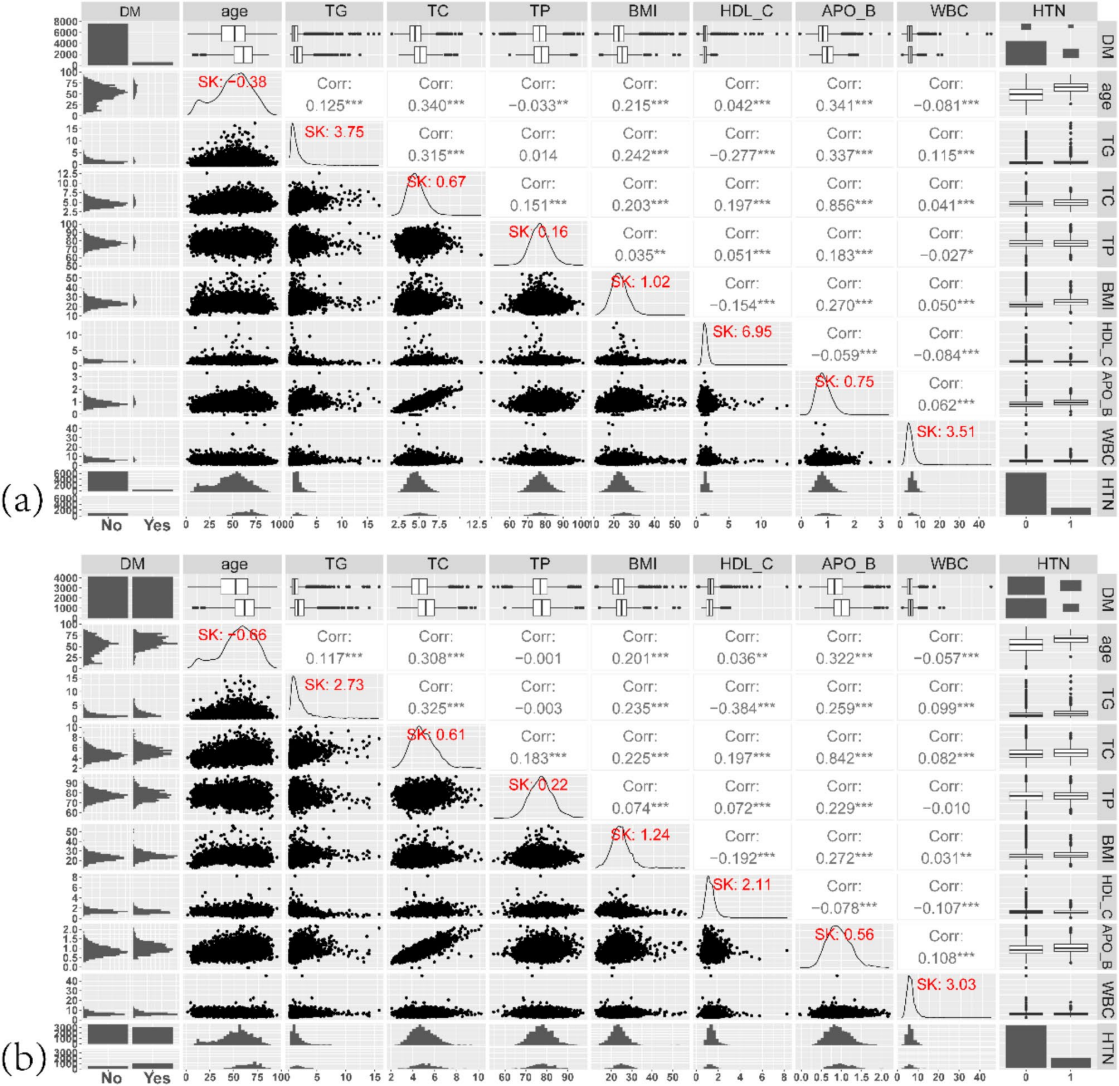
The skewness and correlation between features before and after oversampling the dataset. (**a**) The skewness (SK) and correlation between features before oversampling the dataset; (**b**) The skewness and correlation between features after oversampling the dataset

The process of data analysis based on an interpretable machine-learning method is given in Fig. 1. The random forest data imputation method, which uses a random forest algorithm to predict missing values and fills them into the original data set, deals with missing data. This study used the missForest function of the random forest algorithm to impute missing values in CHNS data. Before oversampling treatment, the data contained 7687 non-pre-diabetic cases and 590 pre-diabetic cases; after treatment, data contained 4177 non-pre-diabetic cases and 4100 pre-diabetic cases. We also checked the skewness and correlation of features before and after oversampling the dataset, as shown in Fig. 2.

#### Training set

The CHNS dataset has a class imbalance, and Random Over-Sampling Examples (ROSE) is a random over-sampling method for dealing with imbalanced data. It can generate artificial samples in the feature space neighborhood of the minority class by using the bootstrap method. It can help improve the performance of classifiers in binary problems where rare classes exist [30]. In this study, the training set data used ROSE to over-sample the minority class of the entire sample. After over-sampling, the ratio of pre-diabetes to non-pre-diabetes was 1:1, and the data was split by using a 5-fold cross-validation method. Four of the subsets were used as training sets (including 6621 cases) to train the model.

#### Test set

One of the subsets was used as a test set to evaluate the model performance (including 1656 cases).

#### External validation dataset

The National Health and Nutrition Examination Survey (NHANES) is a research program aimed at assessing the health and nutritional status of adults and children in the United States, which has been continuous since 1999. These data are published by the National Center for Health Statistics [31]. In this study, we selected 2,188 cases from the 2015–2016 China Health and Nutrition Survey database as validation objects to explore the effectiveness and reliability of the model on external data.

### Predictors identification

The Least Absolute Shrinkage and Selection Operator (LASSO) regression was used for feature selection of the training set to construct the prediction model. During the feature selection process, candidate variables with non-zero coefficients are selected as potential predictors. Recursive feature elimination (RFE) is a recently developed feature selection method for small sample classification problems [32]. Maximum relevance minimum redundancy (mRMR) is a forward selection supervised filtering method that utilizes mutual information as a dependency measure and has been widely applied in various fields such as bioinformatics and omics analysis [33]. We employ LASSO regression, RFE, and mRMR to screen all candidate variables for potential features of the predictive model.

### Machine learning prediction model

#### XGBboost

XGBoost was proposed by Chen and Guestrin in 2016 for developing predictive models [34], using the negative gradient of the loss function as the residual of the current fit to achieve accurate classification. XGBoost performs a second-order Taylor expansion on the loss function and adds a canonical term to balance the decline in the loss function and the complexity of the model, thus reducing the overfitting of the model.

#### Random forest

Random forest (RF) is a commonly used machine learning algorithm that combines the output of multiple decision trees to reach a single result. A forest is established randomly. There are many decision trees in the forest and there is no connection between each tree, then voting to form a strong classifier [35].

#### Support vector machine

Support Vector Machines (SVM) is a binary classification algorithm. Its learning strategy is to maximize the margin. The maximum margin makes it different from the perceptron. It can be formalized as a problem of solving convex quadratic programming, which is also equivalent to the minimization of the regularized hinge loss function [36]. SVM includes kernel techniques, which makes it a nonlinear classifier in essence.

#### Naive Bayes

Naive Bayes (NB) is a deep learning algorithm that utilizes Bayes’ rule together with a strong assumption that the attributes are conditionally independent of the given class. While this independence assumption is often violated in practice, naive Bayes nonetheless often delivers competitive classification accuracy [37].

#### Decision tree

Since introduced in the 1960’s, decision trees (DT) are one of the most effective methods for data mining. This method classifies a population into branch-like segments that construct an inverted tree with a root node, internal nodes, and leaf nodes. The algorithm is non-parametric and can efficiently deal with large, complicated datasets without imposing a complicated parametric structure [38].

#### Artificial neural networks

Artificial neural networks (ANNs) are mathematical models that are based on biological neural networks and composed of interconnected groups of artificial neurons. ANNs belong to the backpropagation class of neural networks, a group of models that uses training methods to minimize errors [39].

#### Model performance evaluation

Test set data was used to evaluate model performance in this study. The area under receiver operating characteristic (AUC), accuracy, sensitivity, specificity, and F1 score were used to assess classification performance. The accuracy represents the degree of agreement between the measured value and the actual value. The sensitivity represents the percentage of the actual disease that is correctly judged as positive by the machine learning method. The specificity represents the percentage of the actual disease that is correctly judged as negative by the machine learning method. With true positive rate (sensitivity) as ordinate and false positive rate (1-specificity) as abscissa, the receiver operating characteristic (ROC) curve is drawn, which shows the overall performance of the binary classifier system. The F1 score is an index used to measure the accuracy of binary classification models in statistics. It considers both the accuracy and recall of the classification model. The F1 score can be regarded as a harmonic mean of the model’s precision and recall, with a maximum of 1 and a minimum of 0.

1 $$\:\text{S}\text{e}\text{n}\text{s}\text{i}\text{t}\text{i}\text{v}\text{i}\text{t}\text{y}\:\left(\text{S}\text{E}\text{N}\right)=\text{T}\text{P}/\left(\text{T}\text{P}+\text{F}\text{N}\right)$$

2 $$\:\text{S}\text{e}\text{n}\text{s}\text{i}\text{t}\text{i}\text{v}\text{i}\text{t}\text{y}\:\left(\text{S}\text{P}\text{E}\right)=\text{T}\text{N}/\left(\text{F}\text{P}+\text{T}\text{N}\right)$$

3 $$\:\text{A}\text{c}\text{c}\text{u}\text{r}\text{a}\text{c}\text{y}\:\left(\text{A}\text{C}\text{C}\right)=\left(\text{T}\text{P}+\text{T}\text{N}\right)/\left(\text{T}\text{P}+\text{F}\text{P}+\text{T}\text{N}+\text{F}\text{N}\right)$$

4 $$\:F1=2\:\frac{precision\cdot\:recall}{precision+recall}$$

TP, TN, FP and FN represent true positive, true negative, false positive and false negative, respectively. Finally, DeLong test [40] was used to compare the statistical significance of the difference between the areas under ROC curves.

#### Interpretable machine learning method

Interpretability is a useful debugging tool for detecting bias in machine learning models. SHapley Additive exPlanations (SHAP) proposed by Lundberg and Lee [41] is a method to explain individual predictions [42]. The Shapley value $\:{\phi\:}_{j}$ is the average marginal contribution of a feature value across all possible coalitions:

5 $$\begin{aligned}&{\phi\:}_{j}\left(val\right)={\sum\:}_{S\subseteq\:\{1,\ldots,p\}\backslash\:\left\{j\right\}}\frac{\left|S\right|!(p-\left|S\right|-1)!}{p!}\cr&\quad\quad\quad\quad\quad\left(val\right(S\cup\:\left\{j\right\})-val(S\left)\right)\cr&va{l}_{x}\left(S\right)=\int\:\widehat{f}({x}_{1},\ldots,{x}_{p})d{P}_{x\notin\:S}-{E}_{X}\left(\widehat{f}\left(X\right)\right)\end{aligned}$$

where *S* is a subset of the features used in the prediction models, $\:x$is the vector of feature values of the instance to be explained, and *p* is the number of features, *val*_*x*_(*S*) is the prediction for feature values in the set *S* that are marginalized over features that are not included in the set *S*.

We proposed an interpretable machine learning method based on the machine learning model with high prediction accuracy, and quantitatively evaluate the contribution of factors on the risk of pre-diabetes. As shown in Fig. 1, the process of this method was listed. First, the data preprocessing stage includes data cleaning and feature selection. We applied the ROSE method to balance the data and then split the data into five folds using cross-validation. Four of the subsets were used as training sets. Then, using the labeled training data, we trained predictive models using RF, SVM, DT, ANNs, Naive Bayes, XGBoost and LR algorithms and estimated their hyperparameters. We also split the original data into five folds using cross-validation and used one of them as a test set. We compared the predictive performance of these classifiers, RF, SVM, DT, ANNs, Naive Bayes, XGBoost and LR models, and used five-fold cross-validation to determine the best predictive model. Next, by using the SHAP method based on the optimal predictive model, we quantified the impact and importance of potential risk factors on pre-diabetes prediction.

#### Parameters adjustment

We used the 5-fold cross-validation method to evaluate each set of hyperparameters. We also compared the average performance metrics of different hyperparameter combinations and selected the optimal one as the final result. We applied this method to select the best parameters for the six models mentioned above. The estimation results of hyperparameters are given in Tables S2–S7.

### Statistical software

All statistical analyses were carried out using R software (version 4.3.0) with packages of “missForest”, “xgboost”, “randomForest”, “e1071”, “SHAPforxgboost”, “pROC”, “caret”, “tidyverse”, “lattice”, “ElemStatLearn”, “ROSE”, “rpart”, “neuralnet”, “caret”, “ggplot2”, and “glmnet”. All *P*-values were two-sided, and statistical significance was claimed when *P* < 0.05.

## Results

### Variable assignment and description

A total of 8,277 individuals including 590 pre-diabetes patients and 7687 non-pre-diabetes patients, and 30 variables were extracted. The following variables were obtained: demography, biochemical indicators, income, lifestyles, energy intake, physical indicators, urbanization, and hypertension.

### Features selected

For feature selection, we employed three methods, LASSO, RFE and mRMR. By comparing the performance of these methods in model construction, we found that LASSO regression outperformed others in terms of accuracy, precision, AUC, and F1 score (Fig S2, Tables S8–S10). Therefore, we chose LASSO regression as the feature selection method, effectively reducing the dimensionality of the data while avoiding overfitting. It is worth noting that the concentration of glucose or glycosylated hemoglobin (HbA1c) are used to define the outcome of pre- diabetes, and these two indices have high correlation with pre-diabetes, which may result in a statistical bias of the other predictors, so we excluded these two indices from the candidate set of predictors.

**Fig. 3:**
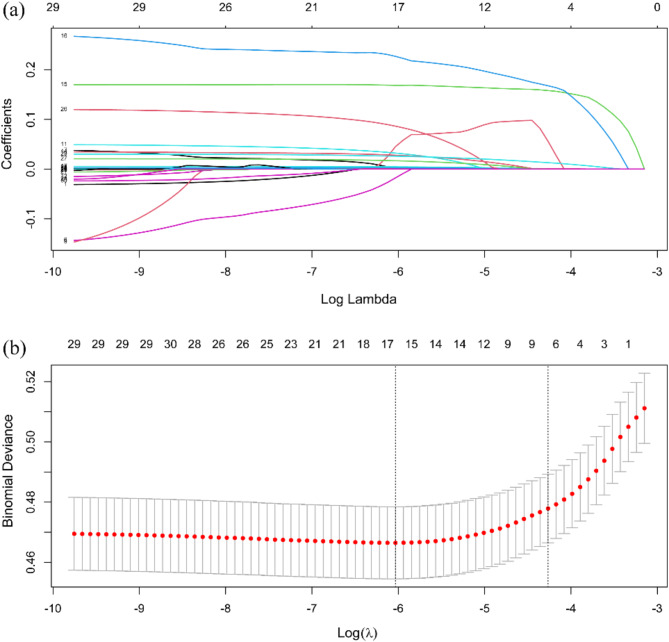
Variable selection by the LASSO binary logistic regression model. (**a**) The process of screening the most suitable value of parameter λ by 10-fold cross-validation in the LASSO model. When log(λ) = -6.1256, the selected parameters are 17, and the LASSO regression model is the optimal. (**b**) The variation characteristics of the variable coefficients

**Fig. 4:**
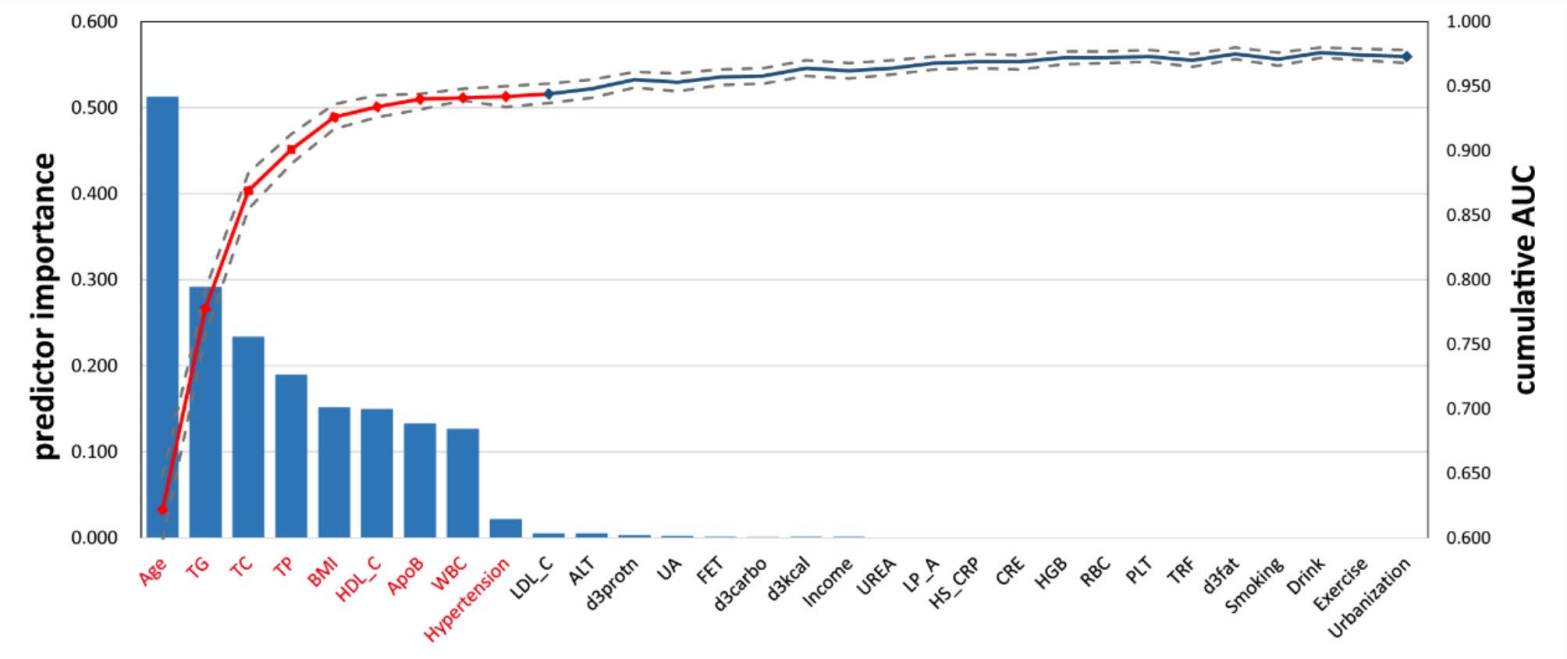
Sequential forward selection from pre-selected candidate predictors. The bar chart represents the importance of the ranked predictors, indicating their contribution to the model classification. The line graph depicts the cumulative AUC (right axis) for each iteration containing one predictor. The top nine predictors (indicated in red) were finally selected for machine learning model constructions

Subsequently, guided by LASSO regression, we initially selected the 17 most important features (Table 1; Fig. 3), then sequentially added these features to the XGBoost classifier based on their importance. We observed that after incorporating the first 9 most important predictive factors, the model’s performance did not significantly improve with the addition of more predictors (Fig. 4). Therefore, we decided to only select these 9 features with non-zero coefficients as predictors for the model.

These 9 selected predictors included: age, total cholesterol (TC), total protein (TP), white blood cell count (WBC), high-density lipoprotein cholesterol (HDL_C), Apo lipoprotein B (ApoB), triglycerides (TG), hypertension, and body mass index (BMI). These variables, as independent variables, together with the occurrence of prediabetes (yes or no) as dependent variables, formed our predictive model. Briefly, our model utilizes nine important features to predict the risk of prediabetes, identified through LASSO regression as having non-zero coefficients, without the need for further predictive factors for model performance. This selection helps maintain the simplicity and effectiveness of the model.

**Table 1:** Least absolute shrinkage and selection operator (LASSO) regression coefficients

Predictors	Coefficient
**TC**	0.22672290
**TG**	0.16844170
**Hypertension**	0.08631334
**APO_B**	0.04510569
**WBC**	0.03212868
**TP**	0.02777698
**Age**	0.02582918
**HDL_C**	-0.01779212
**BMI**	0.01670100
ALT	0.00446703
d3protn	0.00241984
UA	0.00122611
LDL_C	0.00111705
FET	0.00046701
d3carbo	0.00029819
d3kcal	0.00002223
Income	0.00001172

### Comparison of the model performance

**Fig. 5:**
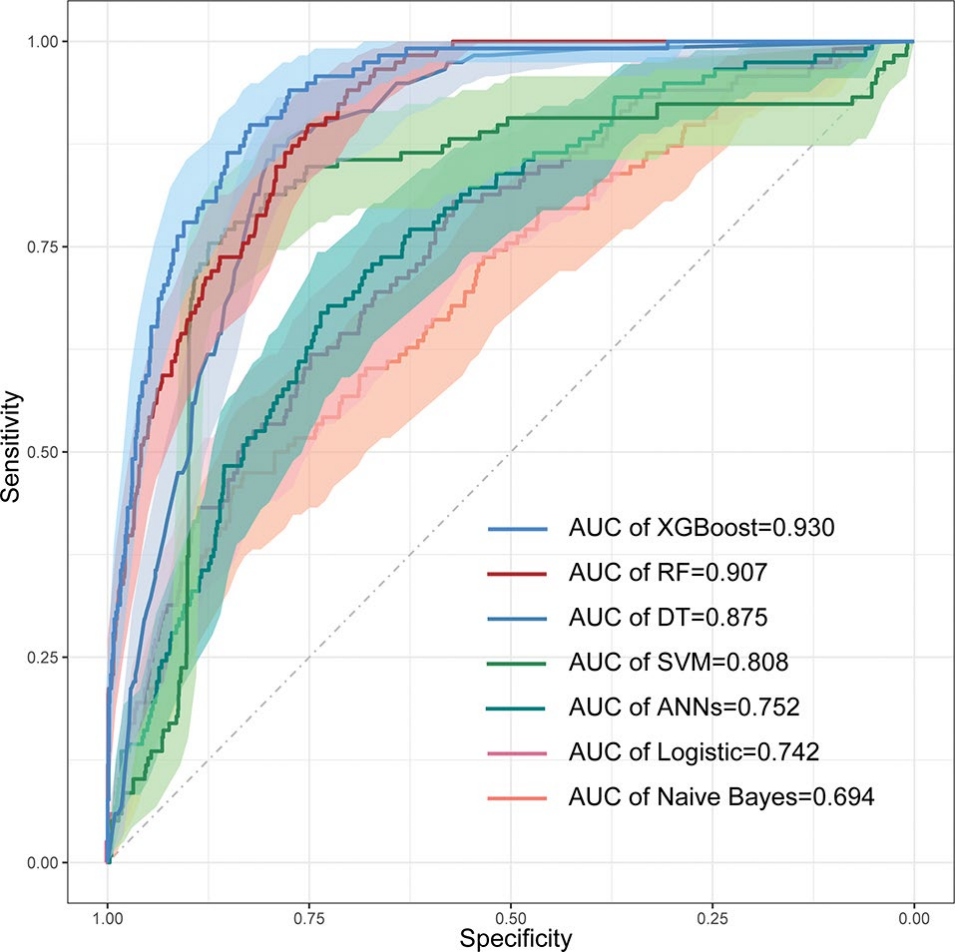
Performance of the machine learning models. ROC curve of the test set. LR, Logistic regression; RF, Random Forest; SVM, Support Vector Machine; XGBoost, eXtreme Gradient Boosting; DT, decision trees; ANNs, artificial neural networks; AUC, the area under the curve under the characteristics of the subjects

Based on the LASSO regression algorithm, nine features with pre-diabetes non-zero coefficients were selected and the best machine learning algorithm was established using the balanced data. XGBoost, random forest (RF), support vector machine (SVM), naive Bayes (NB), decision trees (DT), artificial neural networks (ANNs), and logistic regression (LR) models were constructed, respectively. The accuracy, sensitivity, specificity, AUC, and F1 score of the performance evaluation in training and test and external validation sets are shown in Tables 2, 3 and 4; Fig. 5, respectively.

**Table 2:** Performance of prediction models in training and test sets

Method	Training					Test				
	ACC	SEN	SPE	F1	AUC	ACC	SEN	SPE	F1	AUC
LR	0.686(0.004)	0.696(0.005)	0.675(0.004)	0.687(0.004)	0.737(0.024)	0.929(0.001)	0.02(0.021)	0.998(0.001)	0.048(0.038)	0.75(0.030)
RF	0.862(0.002)	0.887(0.016)	0.838(0.018)	0.864(0.002)	0.947(0.001)	0.797(0.018)	0.837(0.027)	0.794(0.019)	0.371(0.024)	0.91(0.021)
SVM	0.922(0.002)	0.908(0.023)	0.935(0.022)	0.92(0.003)	0.969(0.001)	0.901(0.007)	0.081(0.013)	0.964(0.008)	0.105(0.016)	0.825(0.019)
XGB	0.924(0.005)	0.943(0.01)	0.904(0.008)	0.924(0.005)	0.978(0.001)	0.845(0.01)	0.896(0.027)	0.841(0.013)	0.453(0.011)	0.939(0.007)
DT	0.915(0.006)	0.949(0.014)	0.881(0.011)	0.917(0.006)	0.948(0.003)	0.806(0.012)	0.915(0.035)	0.798(0.014)	0.403(0.016)	0.885(0.009)
NB	0.679(0.002)	0.723(0.035)	0.637(0.031)	0.685(0.014)	0.738(0.002)	0.447(0.062)	0.822(0.062)	0.417(0.067)	0.182(0.019)	0.698(0.033)
ANNs	0.707(0.003)	0.713(0.035)	0.702(0.038)	0.707(0.009)	0.769(0.004)	0.737(0.034)	0.625(0.062)	0.746(0.038)	0.254(0.022)	0.741(0.039)

*Note: LR, Logistic regression; RF, Random Forest; SVM, Support Vector Machine; XGB, eXtreme Gradient Boosting; NB, Naive Bayes; DT, decision trees; ANNs, artificial neural networks; AUC, the area under the curve under the characteristics of the subjects. Standard deviations (SD) for these methods are given in parentheses next to the mean value

**Table 3:** Performance of all folds of XGBoost model in the training, test and external validation set

XGB	Training					Test					External validation				
	ACC	SEN	SPE	F1	AUC	ACC	SEN	SPE	F1	AUC	ACC	SEN	SPE	F1	AUC
Fold 1	0.923	0.937	0.908	0.923	0.977	0.849	0.864	0.848	0.45	0.9300	0.810	0.438	0.848	0.300	0.745
Fold 2	0.916	0.931	0.902	0.917	0.976	0.848	0.890	0.845	0.455	0.948	0.816	0.325	0.866	0.247	0.720
Fold 3	0.924	0.956	0.892	0.925	0.978	0.836	0.915	0.830	0.444	0.933	0.818	0.384	0.862	0.281	0.734
Fold 4	0.928	0.942	0.915	0.929	0.979	0.858	0.881	0.856	0.470	0.94	0.799	0.419	0.838	0.279	0.723
Fold 5	0.927	0.949	0.905	0.927	0.979	0.834	0.932	0.826	0.444	0.942	0.797	0.424	0.835	0.279	0.737

*Note: LR, Logistic regression; RF, Random Forest; SVM, Support Vector Machine; XGB, eXtreme Gradient Boosting; NB, Naive Bayes; DT, decision trees; ANNs, artificial neural networks; AUC, the area under the curve under the characteristics of the subjects

**Table 4:** Performance of the prediction models with and without NLR in the external validation set

Method	External validation for prediction model without NLR					External validation for prediction model with NLR				
	ACC	SEN	SPE	F1	AUC	ACC	SEN	SPE	F1	AUC
LR	0.599(0.032)	0.813(0.036)	0.578(0.039)	0.274(0.007)	0.745(0.002)	0.686(0.003)	0.696(0.005)	0.676(0.004)	0.687(0.004)	0.750(0.002)
RF	0.790(0.014)	0.382(0.038)	0.835(0.017)	0.261(0.013)	0.722(0.004)	0.792(0.016)	0.397(0.034)	0.833(0.020)	0.262(0.010)	0.722(0.004)
SVM	0.891(0.009)	0.100(0.035)	0.972(0.013)	0.146(0.033)	0.616(0.011)	0.891(0.005)	0.027(0.016)	0.979(0.007)	0.043(0.023)	0.655(0.004)
XGB	0.815(0.006)	0.376(0.031)	0.859(0.007)	0.274(0.018)	0.733(0.010)	0.808(0.010)	0.398(0.045)	0.850(0.014)	0.277(0.019)	0.732(0.010)
DT	0.756(0.013)	0.377(0.037)	0.794(0.016)	0.223(0.017)	0.562(0.022)	0.756(0.013)	0.377(0.037)	0.794(0.016)	0.223(0.017)	0.562(0.022)
NB	0.479(0.037)	0.742(0.046)	0.451(0.045)	0.209(0.002)	0.641(0.004)	0.479(0.036)	0.762(0.057)	0.451(0.045)	0.209(0.002)	0.662(0.043)
ANNs	0.787(0.025)	0.420(0.082)	0.825(0.035)	0.267(0.022)	0.678(0.011)	0.574(0.053)	0.749(0.035)	0.557(0.062)	0.248(0.015)	0.691(0.015)

*Note: LR, Logistic regression; RF, Random Forest; SVM, Support Vector Machine; XGB, eXtreme Gradient Boosting; NB, Naive Bayes; DT, decision trees; ANNs, artificial neural networks; AUC, the area under the curve under the characteristics of the subjects. These methods’ standard deviations (SD) are given in parentheses next to the mean value. NLR: neutrophil-to-lymphocyte ratio

**Table 5:** The AUC and DeLong test of the comparison of XGBoost and the other models

	NB	LR	ANNs	SVM	DT	RF	XGBoost
AUC (95% CI)	0.694 (0.644,0.744)	0.742 (0.696,0.788)	0.752 (0.709,0.795)	0.808 (0.761,0.855)	0.875 (0.850,0.900)	0.907 (0.888,0.929)	0.930 (0.915,0.953)
DeLong test (P value)	4.212e-05	2.2e-16	1.996e-13	3.876e-08	5.717e-06	0.0004375	Ref.

*Note: The P values are given in cells and black Bold represents the statistical significance of the difference compared with XGBoost. Abbreviated interpretation: LR, Logistic regression; RF, Random Forest; SVM, Support Vector Machine; XGBoost, eXtreme Gradient Boosting; NB, Naive Bayes; DT, decision trees; ANNs, artificial neural networks. 95%CI: 95% confidence interval

**Fig. 6:**
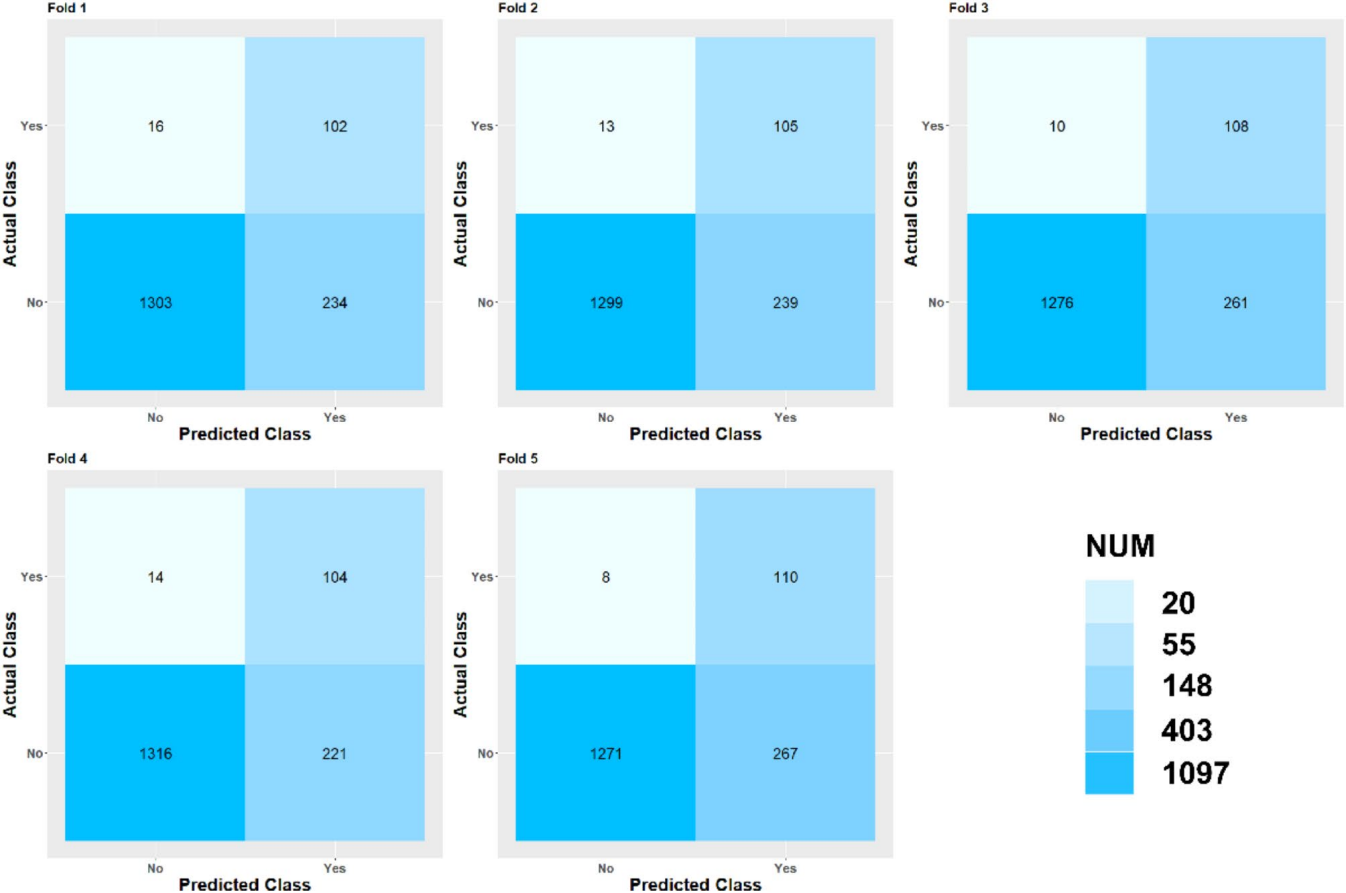
The confusion matrix of all folds of the XGBoost

Table 2 presents the summary of prediction performance for XGBoost, SVM, RF, NB, DT, ANNs and LR. The accuracy (0.929) of Logistic regression was the highest, but its sensitivity (0.021) is lower than other algorithms in the test set. The Random Forest model performed a moderate and reasonable accuracy (0.797), sensitivity (0.837), specificity (0.794), and AUC (0.910) in the test set, its performance on the external validation dataset is also mediocre. Decision trees also performed a moderate and reasonable result in the test set, the accuracy, sensitivity, specificity, F1 and AUC were 0.806, 0.915, 0.789, 0.403 and 0.885. The performance of Naive Bayes is the worst, with an accuracy of only 0.447 and an AUC of 0.698. Table S11 shows the performance of all folds of all models (XGBoost, SVM, RF, NB, DT, ANNs and LR), the confusion matrix of all folds of the XGBoost model is shown in Fig. 6. And we also provided the runtime of all models in Table S13, the training time of the XGBoost model is only 1.56 s, which indicates that the model has a greater advantage. In general, the XGBoost model provided the highest performance in both the training and test sets, the XGBoost model performed best in the test set with the highest AUC (0.939), the accuracy, sensitivity, specificity, and F1 were 0.845, 0.896, 0.841 and 0.453, respectively. Table 3 gives the performance of all folds of the XGBoost model in training, test and external validation sets, respectively. Additionally, we use the NHANES as an external validation set, and carry out two prediction models with and without neutrophil-to-lymphocyte ratio (NLR), respectively. As shown in Table 4, the prediction models with NLR has a slightly improvement of the predictive performance than these models without NLR. This result indicates that integrated into more biomarkers such as NLR may increase the prediction accuracy to some extent. In the DeLong test, all P-values are less than 0.05, implying that these results are statistically significant. Among all, the performance of the XGBoost model was significantly different from the LR, RF, SVM, DT, ANNs and Naive Bayes models, and has a relatively best predictive performance (Table 5), so we consider it as the optimal model in this study, which was an effective classifier for evaluating the prediction model for the risk of pre-diabetes.

### Evaluation of influencing factors of pre-diabetes

**Fig. 7:**
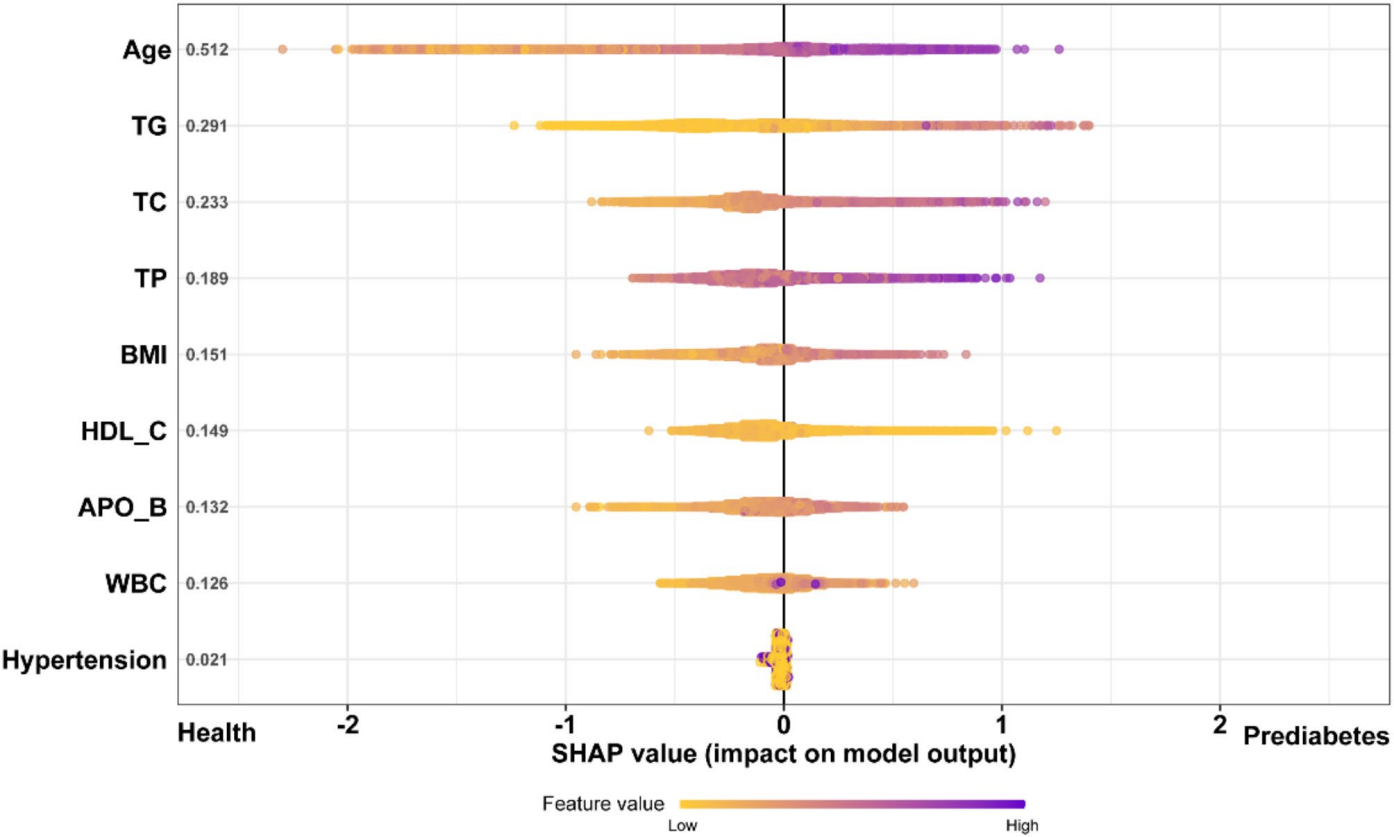
SHAP summary diagram of XGBoost model. The higher the shape value of the feature, the higher the risk of diabetes. Each patient’s contribution to the model for each feature corresponds to a point. Points are colored according to eigenvalues. Purple represents higher eigenvalues and yellow represents lower eigenvalues. The higher the shape value of the feature, the greater the possibility of diabetes. SHAP: Shapley Additive exPlanation

To better determine the importance of each feature for the pre-diabetes prediction model, this study constructed a SHAP summary for the XGBoost model (Fig. 7). The feature importance ranking (Y-axis) indicates the importance of risk factors on the predictive model, and the SHAP value (X-axis) is an index that responds to the influence of a certain feature in the model. Age, TG, and TC were at the top of the ranking list, as shown in the SHAP chart in Fig. 7. The higher the shape value of the feature, the greater the contribution to the risk of pre-diabetes. The width of the range of horizontal bars can be interpreted as the impact on the model prediction that the wider its range, the larger its impact. Purple dots represent higher eigenvalues, and yellow dots represent lower eigenvalues. High values of age and TC correspond to SHAP values greater than zero, indicating that these features may be important risk factors for pre-diabetes.

**Fig. 8:**
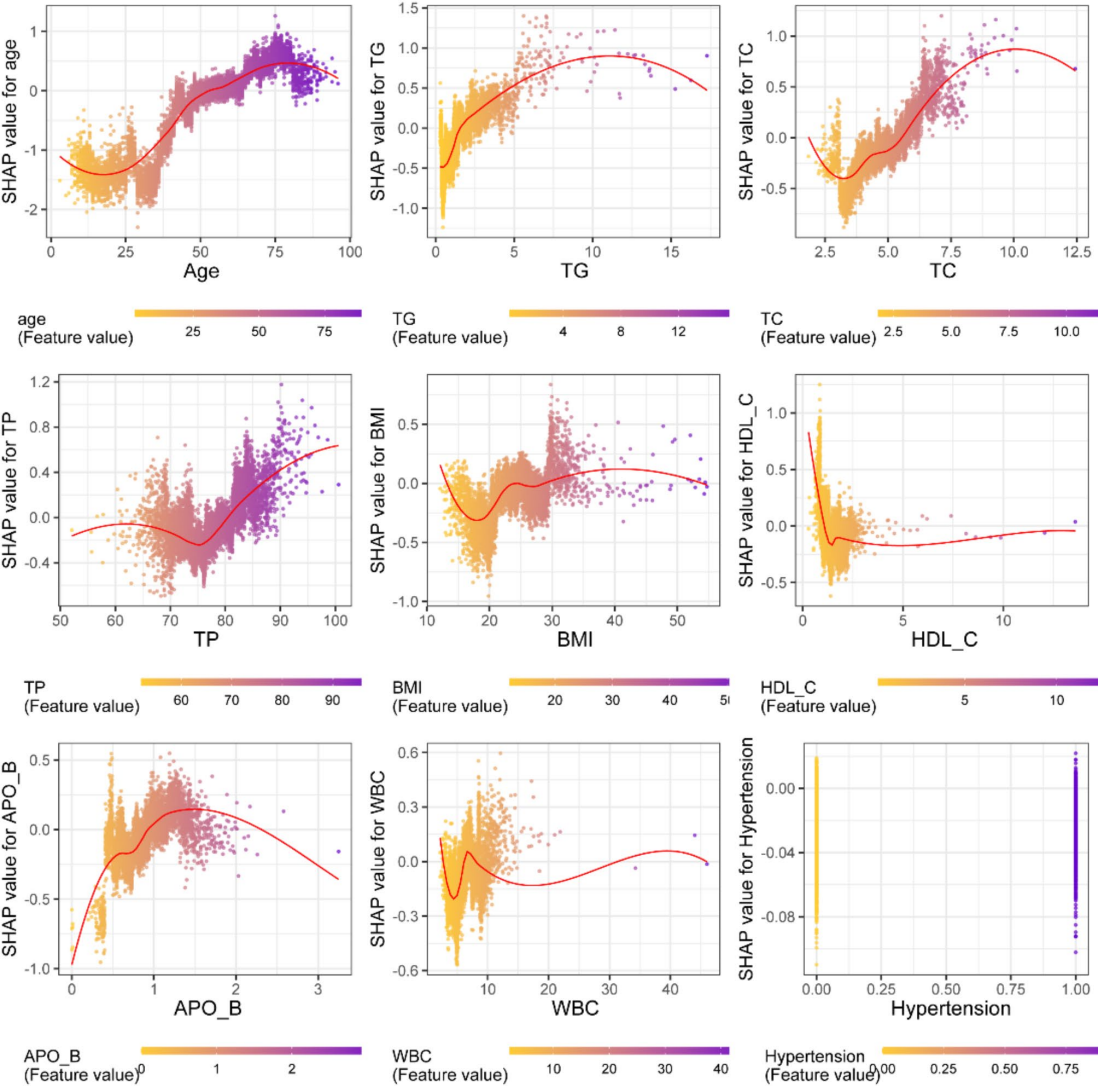
SHAP dependency graph of XGBoost model. The SHAP (Shapley Additive Explanation) value for each trait exceeded zero, indicating an increased risk of diabetes

Figure 8 exhibited the effect of a single feature on the SHAP value based on the XGBoost prediction model of pre-diabetes. It combines SHAP values and each feature value for the plot. When the SHAP value for each feature exceeds zero, it indicates an increased risk of developing pre-diabetes, and vice versa. It can be seen from Fig. 8 that if age greater than 53 years, then the SHAP value increase significantly, which implies that this the individuals age greater than 53 years has a higher risk of pre diabetes. BMI greater than 25, can be defined as an overweight status, has a positive effect on the incidence of pre diabetes. For the biochemical indices, existing studies indicated that TC ranges from 5.2 to 5.7mmol/L represents the slightly elevated lipid. Our result revealed that TC greater than 5.6 mmol /L has a markedly adverse effect on the risk of pre diabetes, which given a more detailed cutoff value to take early action. Additionally, TG below than 1.7 mmol/L are generally considered normal lipid metabolic status, we found that TG greater than 1.4 mmol /L is closely associated with the increase of risk of prediabetes. ApoB in normal population ranges from 0.80 to 1.10 g/L, and we observed from Fig. 8 that ApoB ranged from 0.9 to 2.3 g/L is a wider range for prediabetes risk. Similarly, HDL_C lower than 1.2 mmol/L (normal range 1.16 to 1.42 mmol/L), WBC greater than 6.2 × 10^9^ /L(normal range 4 to 10 × 10^9^L), TP greater than 81 g/L(normal range 63 to 85 g/L) are cutoff values fall in the normal ranges. These results suggested that direct use of existing normal range of biochemical indicators may not be the well predictors of prediabetes risk. To sum up, age greater than 53 years, BMI greater than 25, TC greater than 5.6 mmol /L (100.8 mg/dl), ApoB value greater than 0.9 g/L, TG greater than 1.4 mmol /L(124.2 mg/dl), hypertension, TP greater than 81 g/L, HDL_C lower than 1.2 mmol /L(46.4 mg/dl), WBC greater than 6.2 × 10^9^ /L may be the risk factors for pre-diabetes. This subgroup determined by these cutoff values should be given more attention to early take prevention measures in community and clinic screening program.

### Prediction of the risk of individuals pre-diabetes

**Fig. 9:**
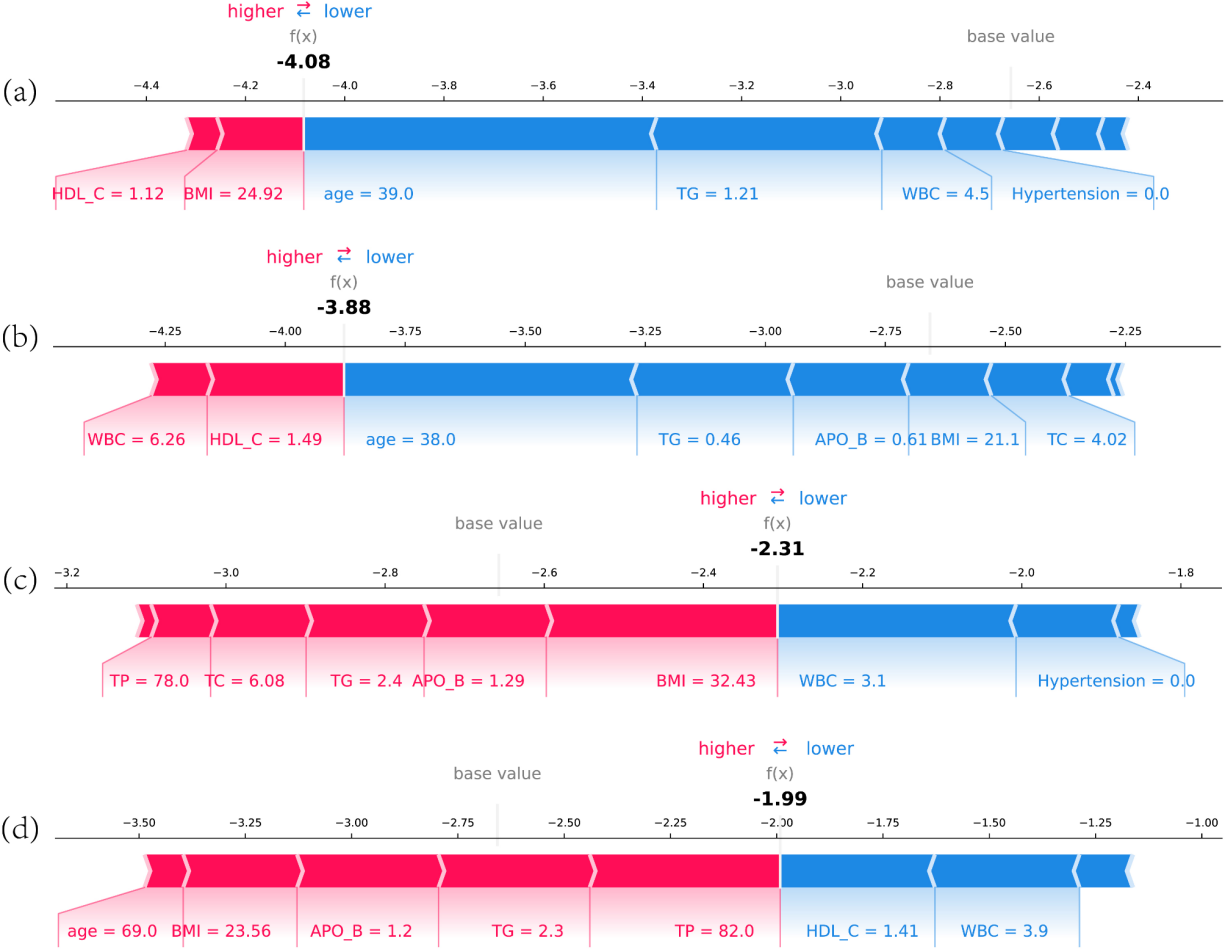
Shapley Additive exPlanation force plot for pre-diabetes patient and health individual. (**a**) and (**b**) are health individuals, (**c**) and (**d**) are pre-diabetes patients

The application of the prediction model to predict the the risk of individuls pre-diabetes is shown in Fig. 9. Four individuals have predicted the risk of pre-diabetes according to their nine selected features. The red area implied that the feature value increases the probability of pre-diabetes and the blue area indicated that the feature value decreases the probability of pre-diabetes. The function *f*(*x*) indicates the comprehensive SHAP value of each individuals. The base value denoted the average SHAP value of all samples. If the value of *f*(*x*) is greater than the base value, the model will predict that the patient may have pre-diabetes. Subplots (a) and (b) showed that a health person could be predicted as not suffering from pre-diabetes. Subplot (c) and (d) presented that a pre-diabetes patient was predicted to suffer from pre-diabetes. Furthermore, a force plot of interpretation for these cases in the internal validation is illustrated in Fig S1. The x-axis represents each patient, while the y-axis represents the contribution of the features. The SHAP force plot aggregates these SHAP values for each observation and demonstrates how the final output was the sum of each predictor attribute. Thus, the XGBoost model can provide a good distinction prediction between pre-diabetes and non-pre-diabetes and indicate the precise risk probabilities according to the individualized circumstances.

## Discussion

Many individuals exhibit impaired fasting glucose, a precursor to diabetes mellitus, often without symptomatic manifestation, thereby remaining unaware of their diabetic condition. Existing evidence suggests that early identification and treatment of pre-diabetes can mitigate its progression to Type 2 Diabetes Mellitus (T2DM). Thus, early prediction of the risk of pre-diabetes has become a key public health issue. Using machine learning to provide a more precise pre-diabetes prediction model is an effective method to deal with multiple attributes of data. Machine learning(ML) can also identify features and patterns related to pre-diabetes by analyzing extensive medical data, such as physical examinations, genetic predispositions, lifestyle factors, etc., and evaluate the risk and possibility of an individual getting the disease based on these features and patterns. Recent studies have corroborated the effectiveness and applicability of ML in prediabetes prediction [25, 26, 43]. There are numerous factors affecting the development of pre-diabetes, and a more precise prediction model is essential to identify and quantify the impacts of these risk factors accurately. In this study, based on the data from the China Health and Nutrition Survey database, we utilized the LASSO regression algorithm to distill nine crucial features from a pool of 30 variables, including age, hypertension, TP, triglyceride, BMI, total cholesterol, WBC, apolipoprotein B and HDL_C. We employed the XGBoost algorithm, an interpretable ML method, which outperformed alternative models such as support vector machines (SVM), random forests (RF), decision trees (DT), artificial neural networks (ANN), naive Bayes, and logistic regression (LR) in terms of predictive accuracy. Additionally, we utilized SHAP values to quantify the influence and significance of each potential risk factor on prediabetes. It is noteworthy that our model is solely based on nine easily accessible predictors associated with pre-diabetes, which can be conveniently collected from physical measures, and simple blood tests. Thus, the prediction model may be widely used in practice.

In our prediction model, age is the most important factor in pre-diabetes. Previous studies have shown that age is a risk factor for the development of diabetes [11, 44, 45]. The dysregulation of the cell cycle due to cellular senescence and the consequent proliferative arrest of beta cells with aging is of relevance, and the loss or dysfunction of pancreatic beta cells plays a crucial role in the pathogenesis of type 2 diabetes. Compared with other studies, this study marked the cutoff value of age. The SHAP showed that age greater than 53 years may be the most important risk factor for pre-diabetes. In the case of obesity, the body produces large amounts of insulin that can lead to a reduction in the function of β cells to produce insulin, further leading to insulin resistance (IR) [46], and high BMI also leads to a high prevalence of pre-diabetes [47]. Pre-diabetes is significantly associated with the future development of cardiovascular diseases [48]. Thus, regularly screening individuals over 53 years old for prediabetes and maintaining a healthy lifestyle to reduce BMI may help prevent the progression from prediabetes to T2DM.

Existing evidence demonstrated that IR is the mechanism for the development of T2DM. IR also may block the secretion of apolipoprotein B (ApoB) contributing to the accumulation of cellular TG [47]. It was found that the proportion of low HDL-C and high TG was alarmingly high among the population with pre-diabetes in rural Bangladesh [49], which is consistent with the results of this study. High triglyceride levels may worsen glucose metabolism. Studies have shown that high concentrations of free fatty acids cause an increased likelihood of IR [50, 51]. IR not only gives rise to metabolic abnormalities but also predisposes to cardiovascular-related diseases such as hypertension and vascular stiffness, in turn, excessive arterial stiffness and impaired vasodilation affect IR and aggravate diabetes [52]. Meanwhile, ApoB plays a key role in promoting atherosclerosis [53]. A cross-sectional study further demonstrated that serum TC was associated with β-cell dysfunction in subjects with normal glucose tolerance [54], In the study of Alqahtani et al. [55], the prevalence of high TC was significantly higher in the pre-diabetic group. The results of these two studies are consistent with the present study. Biomarkers showed that the dynamics of normal blood glucose varied from prediabetic to diabetic subjects, with a gradual increase in WBC [56], thus suggesting that WBC could be a predictor of pre-diabetes. Compared to other studies, this study quantifies the bounds of risk factors by using the Shapley Additive exPlanation method and shows the effect of individual features on the output of the XGBoost model. When age is over 53 years old, TG is greater than 1.4 mmol/L (124.2 mg/dl), TC is greater than 5.6 mmol/L (100.8 mg/dl), TP is greater than 81 g/L or too low, BMI is greater than 25, HDL_C is less than 1.2 mmol/L (46.4 mg/dl), ApoB value is greater than 0.9 g/L, and WBC is greater than 6.2 × 10^9^ /L or lower than 2.6 × 10^9^ /L, hypertension may be a risk factor for prediabetes. In addition, this study also showed that both high and low total protein are risk factors for pre-diabetes. A possible reason may be that prediabetic patients have higher protein loss during urination, resulting in lower total protein levels in prediabetic patients [57]. The relationship between total protein and pre-diabetes is a complex topic, and there is no clear evidence to prove that high total protein levels may cause the occurrence of pre-diabetes.

XGBoost algorithm is an integrated algorithm based on the decision tree, and it is a non-parametric estimation whose correlation of independent variables has no significant effect on the model [12]. Earlier studies have shown that Logistic regression models are usually used to assess the risk of diabetes. Li et al. established a risk prediction model for T2DM based on genotyping results to predict the risk of T2DM in Northern China [58]. However, since logistic regression is a linear model and sensitive to multicollinearity data, the high correlation between independent variables will distort the weight parameter estimation of the model, so it has great limitations and may incorporate unnecessary features into the model. The machine learning algorithm has a marked advantage in the prediction of pre-diabetes, the correlation of independent variables has no significant impact on the model. When the sample size and dimension of the data set are large, the XGBoost algorithm has advantages over the logistic regression algorithm. In our study, RF performed better after integration, but still not as well as XGBoost. SVM was less effective in classification prediction, probably because the SVM algorithm is difficult to implement for large-scale training samples. The naive Bayes algorithm performed the worst in this classification prediction, perhaps because it is more difficult to handle continuous features. Although Naive Bayes does not perform well, all other algorithms perform better than the traditional binary logistic algorithm and show good performance, some machine learning algorithms such as Artificial Neural Networks, Random Forests, Support Vector Machines, Decision Trees, etc. have better performance than these traditional methods in prediction of pre-diabetes. Among these machine learning algorithms, XGBoost performs best, with the highest AUC (0.939) and F1 Scour (0.453) in test sets, which are the key indicators for evaluating the function of the prediction model. Its accuracy, sensitivity, specificity and AUC tend to be stable and provide the best performance in machine learning algorithms, which shows that the XGBoost algorithm has a stronger advantage in processing high-dimensional data. In addition, the SHAP value helps make the output of the XGBoost model visualized and clinically interpretably reasonable. The SHAP method can help determine the most critical risk factor boundaries for pre-diabetes, and provide more targeted recommendations for the treatment of diabetic inpatients and the management of pre-diabetic patients.

However, this study also has some limitations. First of all, this study used existing factors collected from the CHNS and NHANES datasets, however, people in different regions have certain differences in lifestyle, dietary habits, health status, physiological conditions, and so on. The model used in this study is the CHNS data from eastern China, and the external validation uses the NHANES data of the US population, which may limit the applicability of this model, but also provides ideas for subsequent studies on the incidence risk of pre-diabetes in different regions. Thus, further studies that test the prediction model across diverse populations [59] and incorporated circulating metabolites data [60] would present a more validated path to improve the predictive performance. Secondly, T2DM is a complex chronic disease that is closely related to genetic factors. The interaction between genetic susceptibility variants and environmental cues leads to the occurrence of T2DM [61], and lack of genetic factors may increase the error of the prediction model, thus, adding more gene variables into the prediction model may further improve the prediction accuracy of the proposed model. Thirdly, machine learning methods also have their limitation. We tried to employ the machine learning model by interpreting the impact and importance of candidate factors with the SHAP method, however, the process was data-driven and aimed to improve the prediction performance, thus, we paid less attention to the underlying mechanisms and public health reference value [62]. In additon, other biochemical indicators may also have good predictive power for prediabetes, such as Neutrophil-to-lymphocyte ratio (NLR) [63], our result indicated that integrated into more biomarkers such as NLR may increase the prediction accuracy. Thus, considering more biochemical indicators could significantly improve predictive performance. Fourth, recently machine learning with causal inference has become one of the hot spots in machine learning and its applications [64, 65]. For example, Wu et al. [66] conducted propensity score matching to balance the confounders. Lemp et al. using regression discontinuity design to evaluate the effect of behaviour change in a nationwide diabetes prevention programme [67]. Additionally, Mendelian randomisation design, as an extension of instrumental variable analysis, has been used to explore the causal association between adiposity and diabetes [68]. Thus, these causal inference techniques could feasibly be applied to pre-diabetes studies and provide a deeper understanding the underlying mechanisms of pre-diabetes. These issues should be considered in the future.

## Conclusion

In summary, we identified nine easily accessible predictors based on LASSO regression and then employed the LR, RF, SVM, DT, Naive Bayes, ANNs, and XGBoost models to predict the risk of pre-diabetes. Compared to RF, SVM, DT, Naive Bayes, ANNs, and LR models, the XGBoost model exhibited a higher AUC value (0.939). Furthermore, SHAP served as an interpretable visualization method for the output of the XGBoost model, quantifying the importance and effects of candidate predictors for pre-diabetes. SHAP analysis indicated that age over 53 years, BMI over 25, TC over 5.6 mmol /L (100.8 mg/dl), ApoB value over 0.9 g/L, TG over 1.4 mmol /L (124.2 mg/dl), hypertension, TP over 81 g/L, HDL_C less than 1.2 mmol /L (46.4 mg/dl), WBC over 6.2 × 10^9^ /L may be the risk factors for pre-diabetes. Due to regional variations, our results may not be applicable to all populations and ignore genetic influences on pre-diabetes. Nevertheless, our approach compares different machine learning algorithms to find the optimal predictive model for pre-diabetes and leverages the SHAP method for interpretability regarding factors influencing pre-diabetes risk. The results can be used to identify individuals at high risk of pre-diabetes in the general population, providing effective and operability early intervention measures for pre-diabetes.

## Electronic supplementary material

Below is the link to the electronic supplementary material.

Supplementary Material 1

## Data Availability

The data used in this study can be downloaded from the website of CHNS at http://www.cpc.unc.edu/projects/china and NHANES at https://wwwn.cdc.gov/nchs/nhanes/continuousnhanes/default.aspx?BeginYear=2015.

## Abbreviations

T2DM: Type 2 Diabetes Mellitus
XGBoost: Extreme Gradient Boosting
RF: Random Forest
SVM: Support Vector Machine
NB: Naive Bayes
ANNs: Artificial Neural Networks
DT: Decision Trees
LR: Logistic Regression
CHNS: China Health and Nutrition Survey
NHANES: The National Health and Nutrition Examination Survey
SHAP: Shapley Additive Explanation
IFG: Impaired fasting glucose
IGT: Impaired glucose tolerance
GDQS: Global Diet Quality Score
BMI: Body mass index
AUC: The area under receiver operating characteristic
ROSE: Random Over-Sampling Examples
LASSO: The Least Absolute Shrinkage and Selection Operator
RFE: Recursive feature elimination
mRMR: maximum relevance minimum redundancy
ROC: The receiver operating characteristic
TP: True positive
TN: True negative
FP: False positive
FN: False negative
TC: Total cholesterol
TP: Total protein
WBC: White blood cell
HDL_C: High-density lipoprotein cholesterol
ApoB: Apolipoprotein B
TG: Triglycerides
ACC: Accuracy
SEN: Sensitivity
SPE: Specificity
ML: Machine learning
IR: Insulin resistance
NLR: Neutrophil-to-lymphocyte ratio
