# Supplementary material for: Interpretable machine learning method to predict the risk of pre-diabetes using a national-wide cross-sectional data: evidence from CHNS
Source: BMC Public Health. 2025 Mar 26;25:1145. doi: 10.1186/s12889-025-22419-7 (PMC11938594; doi:10.1186/s12889-025-22419-7)
Supplement: Supplementary file 1 — Supplementary Material 1 [file 12889_2025_22419_MOESM1_ESM.docx]

Appendix. Supplementary materials

**Supplementary material**

**Table S1. Variable assignment and description.**

**Table S2. Key parameters of the eXtreme Gradient Boosting (XGBoost) model.**

**Table S3. Key parameters of the Random Forest (RF) model.**

**Table S4. Key parameters of the Support Vector Machines (SVM) model.**

**Table S5. Key parameters of the Decision Trees (DT) model.**

**Table S6. Key parameters of the Artificial Neural Networks (ANNs) model.**

**Table S7. Key parameters of the Naive Bayes (NB) model.**

**Table S8. Screening Results of Recursive Feature Elimination (REF).**

**Table S9. Performance of prediction models of the combinations selected by Recursive feature elimination.**

**Table S10. Performance of prediction models of the combinations selected by Maximum relevance minimum redundancy.**

**Table S11. Performance of all folds of prediction models in the training, test and external validation set.**

**Table S12. The DeLong test compares results across the models.**

**Table S13. Runtime of all models.**

**Fig S1. SHAP force plot.**

**Fig S2. The result of Recursive feature elimination.**


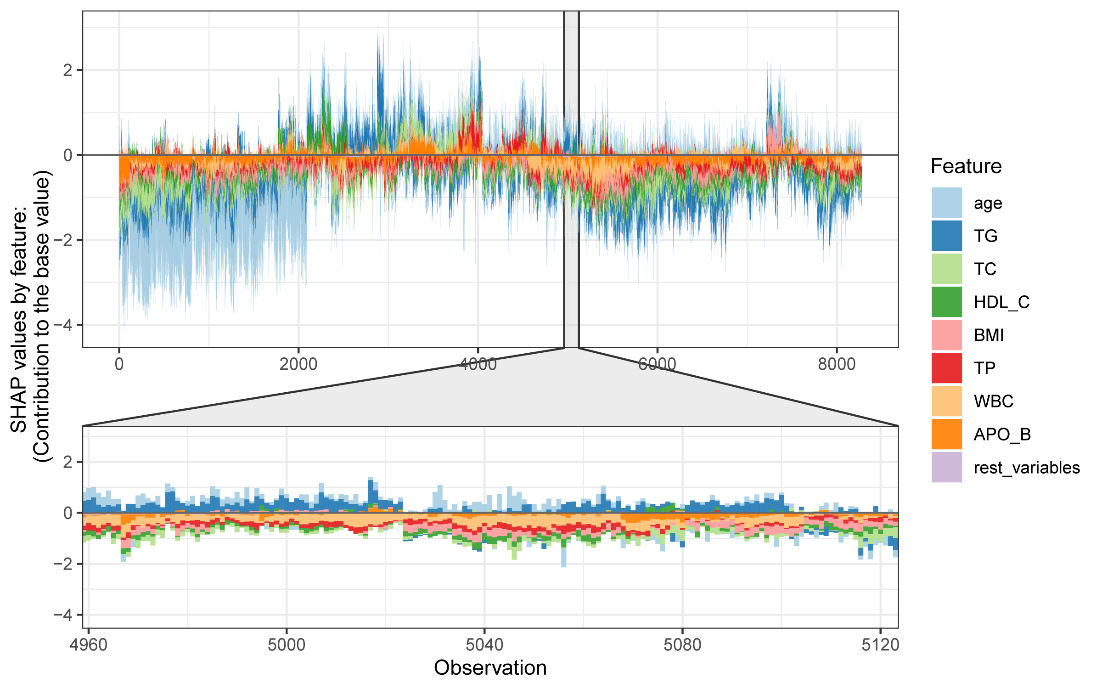


**Fig S1. SHAP force plot.**

The SHAP force plot basically stacks these SHAP values for each observation, and show how the final output was obtained as a sum of each predictor’s attributions.

**Table S1. Variable assignment and description.**

| **variable** | **Description of valuation** |
| --- | --- |
| **Class(Prediabetes)** | No =0, Yes=1 |
| **Smoke** | No=0, Yes=1 |
| **Hypertension** | No=0, Yes=1 |
| **Alcohol intake** | No=0, Yes=1 |
| **Education** | unlettered =0  graduated from primary school =1  lower middle school degree =2  upper middle school degree =3  technical or vocational degree =4  university or college degree =5  master’s degree or higher =6 |
| **Income** | Yuan |
| **d3kcal** | 3-Day Average: total energy (kcal) |
| **d3fat** | 3-Day Average: Fat (g) |
| **d3protein**  **d3carbo** | 3-Day Average: Protein (g)  3-Day Average: Carbohydrate(g) |
| **BMI** | Body Mass Index |
| **Exercise** | Exercise |
| **age** | Age |
| **Urea** | Urea (mmol /L) |
| **UA** | Uric acid (umol /L) |
| **LP_A** | Lipoprotein A-1(mg/L) |
| **HS_CRP** | High-sensitivity CPR (mg/L) |
| **CRE** | Creatinine (umol /L) |
| **LDL_C** | Low-density lipoprotein (mmol /L) |
| **HDL_C** | High-density lipoprotein (mmol /L) |
| **ApoB** | Apolipoprotein B (g/L) |
| **HGB** | Hemoglobin (g /L) |
| **WBC** | White Blood Cell (10^9^ /L) |
| **RBC** | Red Blood Cell (106/L) |
| **PLT** | Platelet (109 /L) |
| **TP** | Total protein (g/L) |
| **ALB** | Albumin (g/L） |
| **TG** | Triglyceride (mmol /L) |
| **TC** | Total cholesterol (mmol /L) |
| **ALT** | Alanine Transaminase (U/L） |
| **TRF**  **NLR** | Transferrin(mg/dl)  neutrophil-to-lymphocyte ratio |

**Table S2. Key parameters of the eXtreme Gradient Boosting (XGBoost) model.**

| **Parameter Name** | **Value** |
| --- | --- |
| eta | 0.05169 |
| gamma | 0.5 |
| max_depth | 7 |
| subsample | 0.81920 |
| colsample_bytree | 0.70069 |
| verbose | 1 |
| min_child_weight | 6 |
| max_delta_step | 7 |
| early_stopping_rounds | 200 |
| seed | 1000 |

**Table S3. Key parameters of the Random Forest (RF) model.**

| **Parameter Name** | **Value** |
| --- | --- |
| ntree | 1000 |
| mtry | 2 |
| maxnodes | 160 |
| sampsize | 5297 |
| seed | 1000 |

**Table S4. Key parameters of the Support Vector Machines (SVM) model.**

| **Parameter Name** | **Value** |
| --- | --- |
| cost | 1 |
| gamma | 0.5 |
| seed | 1000 |

**Table S5. Key parameters of the Decision Trees (DT) model.**

| **Parameter Name** | **Value** |
| --- | --- |
| minsplit | 10 |
| cp | 0.001 |
| seed | 1000 |

**Table S6. Key parameters of the Artificial Neural Networks (ANNs) model.**

| **Parameter Name** | **Value** |
| --- | --- |
| hidden | 5 |
| threshold | 0.1 |
| stepmax | 1e6 |
| seed | 1000 |

**Table S7. Key parameters of the Naive Bayes (NB) model.**

| **Parameter Name** | **Value** |
| --- | --- |
| fL | 1 |
| adjust | 0.5 |
| laplace | 0.01 |
| seed | 1000 |

**Table S8. Screening Results of Recursive Feature Elimination (REF).**

| **Variables** | **Accuracy** | **Kappa** | **AccuracySD** | **KappaSD** |
| --- | --- | --- | --- | --- |
| **age** | 0.928 | 0.004069 | 0.001606 | 0.009098 |
| **TG** | 0.9262 | 0.035446 | 0.001583 | 0.029633 |
| **UA** | 0.9273 | 0.035656 | 0.0009 | 0.011883 |
| **TC** | 0.9263 | 0.02289 | 0.000941 | 0.011005 |
| **APO_B** | 0.9276 | 0.026 | 0.002668 | 0.021975 |
| **FET** | 0.9284 | 0.024601 | 0.001325 | 0.020426 |
| **CRE** | 0.9286 | 0.02787 | 0.00109 | 0.018978 |
| **HDL_C** | 0.9286 | 0.022343 | 0.000899 | 0.017219 |
| **d3protn** | 0.9285 | 0.019399 | 0.001323 | 0.01874 |


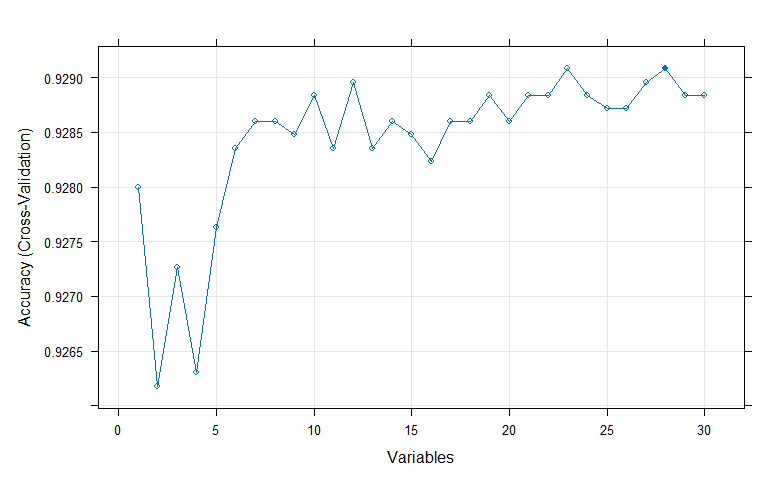


**Fig S2.** **The result of Recursive feature elimination.**

**Table S9. Performance of prediction models of the combinations selected by Recursive feature elimination.**

| **Method** | **Training** | | | | |  | **Test** | | | | |
| --- | --- | --- | --- | --- | --- | --- | --- | --- | --- | --- | --- |
|  | **ACC** | **SEN** | **SPE** | **F1** | **AUC** |  | **ACC** | **SEN** | **SPE** | **F1** | **AUC** |
| **LR** | 0.678 | 0.685 | 0.671 | 0.678 | 0.747 |  | 0.678 | 0.685 | 0.671 | 0.678 | 0.727 |
| **RF** | 0.860 | 0.896 | 0.824 | 0.864 | 0.951 |  | 0.779 | 0.847 | 0.774 | 0.354 | 0.919 |
| **SVM** | 0.711 | 0.766 | 0.658 | 0.724 | 0.778 |  | 0.645 | 0.762 | 0.635 | 0.234 | 0.767 |
| **XGB** | 0.913 | 0.917 | 0.909 | 0.912 | 0.974 |  | 0.836 | 0.881 | 0.832 | 0.434 | 0.931 |
| **DT** | 0.924 | 0.960 | 0.889 | 0.926 | 0.953 |  | 0.695 | 0.695 | 0.720 | 0.693 | 0.767 |
| **NB** | 0.679 | 0.768 | 0.591 | 0.703 | 0.735 |  | 0.589 | 0.762 | 0.576 | 0.209 | 0.767 |
| **ANNs** | 0.632 | 0.928 | 0.341 | 0.714 | 0.672 |  | 0.356 | 0.949 | 0.311 | 0.173 | 0.630 |

LR, Logistic regression; RF, Random Forest; SVM, Support Vector Machine; XGB, eXtreme Gradient Boosting; NB, Naive Bayes; DT, decision trees; ANNs, artificial neural networks; AUC, the area under the curve under the characteristics of the subjects. Standard deviations (SD) for these methods are given in parentheses next to the mean value.

**Table S10. Performance of prediction models of the combinations selected by Maximum relevance minimum redundancy.**

| **Method** | **Training** | | | | |  | **Test** | | | | |
| --- | --- | --- | --- | --- | --- | --- | --- | --- | --- | --- | --- |
|  | **ACC** | **SEN** | **SPE** | **F1** | **AUC** |  | **ACC** | **SEN** | **SPE** | **F1** | **AUC** |
| **LR** | 0.662 | 0.609 | 0.715 | 0.641 | 0.722 |  | 0.827 | 0.016 | 0.997 | 0.032 | 0.71 |
| **RF** | 0.839 | 0.844 | 0.833 | 0.838 | 0.930 |  | 0.782 | 0.762 | 0.784 | 0.333 | 0.868 |
| **SVM** | 0.698 | 0.613 | 0.782 | 0.668 | 0.764 |  | 0.838 | 0.381 | 0.873 | 0.251 | 0.72 |
| **XGB** | 0.907 | 0.910 | 0.903 | 0.906 | 0.970 |  | 0.842 | 0.864 | 0.841 | 0.438 | 0.929 |
| **DT** | 0.918 | 0.952 | 0.886 | 0.920 | 0.953 |  | 0.774 | 0.525 | 0.793 | 0.249 | 0.72 |
| **NB** | 0.649 | 0.589 | 0.707 | 0.625 | 0.682 |  | 0.202 | 0.805 | 0.156 | 0.125 | 0.583 |
| **ANNs** | 0.627 | 0.782 | 0.475 | 0.675 | 0.664 |  | 0.494 | 0.745 | 0.475 | 0.173 | 0.641 |

The screening results of the maximum correlation minimum redundancy method are: TG, LDL_C, HS_CRP, d3protn, Exercise, Hypertension, Urbanization, TP, ET.

**Table S11. Performance of all folds of prediction models in the training, test and external validation set.**

| **Method** | **Training** | | | | |  | **Test** | | | | |  | **External validation** | | | | |
| --- | --- | --- | --- | --- | --- | --- | --- | --- | --- | --- | --- | --- | --- | --- | --- | --- | --- |
|  | **ACC** | **SEN** | **SPE** | **F1** | **AUC** |  | **ACC** | **SEN** | **SPE** | **F1** | **AUC** |  | **ACC** | **SEN** | **SPE** | **F1** | **AUC** |
| **LR** |  |  |  |  |  |  |  |  |  |  |  |  |  |  |  |  |  |
| **Fold 1** | 0.692 | 0.704 | 0.679 | 0.693 | 0.695 |  | 0.928 | 0.008 | 0.999 | 0.017 | 0.742 |  | 0.692 | 0.704 | 0.679 | 0.693 | 0.695 |
| **Fold 2** | 0.686 | 0.697 | 0.675 | 0.687 | 0.746 |  | 0.930 | 0.051 | 0.998 | 0.094 | 0.802 |  | 0.686 | 0.697 | 0.675 | 0.687 | 0.746 |
| **Fold 3** | 0.684 | 0.690 | 0.678 | 0.684 | 0.749 |  | 0.929 | 0.008 | 0.999 | 0.017 | 0.730 |  | 0.684 | 0.690 | 0.678 | 0.684 | 0.749 |
| **Fold 4** | 0.683 | 0.695 | 0.670 | 0.685 | 0.748 |  | 0.929 | 0.034 | 0.997 | 0.063 | 0.749 |  | 0.683 | 0.695 | 0.670 | 0.685 | 0.748 |
| **Fold 5** | 0.685 | 0.696 | 0.674 | 0.686 | 0.749 |  | 0.928 | 0 | 0.999 | NAN | 0.729 |  | 0.685 | 0.696 | 0.674 | 0.686 | 0.749 |
| **RF** |  |  |  |  |  |  |  |  |  |  |  |  |  |  |  |  |  |
| **Fold 1** | 0.860 | 0.894 | 0.829 | 0.864 | 0.948 |  | 0.793 | 0.847 | 0.789 | 0.368 | 0.907 |  | 0.860 | 0.894 | 0.829 | 0.864 | 0.948 |
| **Fold 2** | 0.860 | 0.889 | 0.831 | 0.863 | 0.946 |  | 0.806 | 0.881 | 0.800 | 0.393 | 0.946 |  | 0.860 | 0.889 | 0.831 | 0.863 | 0.946 |
| **Fold 3** | 0.863 | 0.872 | 0.854 | 0.863 | 0.947 |  | 0.796 | 0.814 | 0.794 | 0.362 | 0.891 |  | 0.863 | 0.872 | 0.854 | 0.863 | 0.947 |
| **Fold 4** | 0.865 | 0.870 | 0.861 | 0.865 | 0.946 |  | 0.821 | 0.822 | 0.821 | 0.396 | 0.906 |  | 0.865 | 0.870 | 0.861 | 0.865 | 0.946 |
| **Fold 5** | 0.862 | 0.908 | 0.817 | 0.867 | 0.947 |  | 0.771 | 0.822 | 0.767 | 0.338 | 0.902 |  | 0.862 | 0.908 | 0.817 | 0.867 | 0.947 |
| **SVM** |  |  |  |  |  |  |  |  |  |  |  |  |  |  |  |  |  |
| **Fold 1** | 0.921 | 0.877 | 0.963 | 0.916 | 0.969 |  | 0.912 | 0.085 | 0.975 | 0.120 | 0.808 |  | 0.921 | 0.877 | 0.963 | 0.916 | 0.969 |
| **Fold 2** | 0.921 | 0.917 | 0.925 | 0.920 | 0.967 |  | 0.896 | 0.068 | 0.959 | 0.085 | 0.855 |  | 0.921 | 0.917 | 0.925 | 0.920 | 0.967 |
| **Fold 3** | 0.926 | 0.914 | 0.937 | 0.924 | 0.969 |  | 0.896 | 0.076 | 0.959 | 0.095 | 0.829 |  | 0.926 | 0.914 | 0.937 | 0.924 | 0.969 |
| **Fold 4** | 0.921 | 0.938 | 0.905 | 0.922 | 0.969 |  | 0.895 | 0.102 | 0.956 | 0.122 | 0.808 |  | 0.921 | 0.938 | 0.905 | 0.922 | 0.969 |
| **Fold 5** | 0.922 | 0.896 | 0.947 | 0.919 | 0.971 |  | 0.905 | 0.076 | 0.969 | 0.102 | 0.826 |  | 0.922 | 0.896 | 0.947 | 0.919 | 0.971 |
| **XGB** |  |  |  |  |  |  |  |  |  |  |  |  |  |  |  |  |  |
| **Fold 1** | 0.923 | 0.937 | 0.908 | 0.923 | 0.977 |  | 0.849 | 0.864 | 0.848 | 0.450 | 0.930 |  | 0.923 | 0.937 | 0.908 | 0.923 | 0.977 |
| **Fold 2** | 0.916 | 0.931 | 0.902 | 0.917 | 0.976 |  | 0.848 | 0.890 | 0.845 | 0.455 | 0.948 |  | 0.916 | 0.931 | 0.902 | 0.917 | 0.976 |
| **Fold 3** | 0.924 | 0.956 | 0.892 | 0.925 | 0.978 |  | 0.836 | 0.915 | 0.83 | 0.444 | 0.933 |  | 0.924 | 0.956 | 0.892 | 0.925 | 0.978 |
| **Fold 4** | 0.928 | 0.942 | 0.915 | 0.929 | 0.979 |  | 0.858 | 0.881 | 0.856 | 0.470 | 0.940 |  | 0.928 | 0.942 | 0.915 | 0.929 | 0.979 |
| **Fold 5** | 0.927 | 0.949 | 0.905 | 0.927 | 0.979 |  | 0.834 | 0.932 | 0.826 | 0.444 | 0.942 |  | 0.927 | 0.949 | 0.905 | 0.927 | 0.979 |
| **DT** |  |  |  |  |  |  |  |  |  |  |  |  |  |  |  |  |  |
| **Fold 1** | 0.911 | 0.947 | 0.876 | 0.913 | 0.946 |  | 0.805 | 0.856 | 0.801 | 0.385 | 0.875 |  | 0.911 | 0.947 | 0.876 | 0.913 | 0.946 |
| **Fold 2** | 0.924 | 0.955 | 0.893 | 0.925 | 0.952 |  | 0.821 | 0.915 | 0.813 | 0.421 | 0.882 |  | 0.924 | 0.955 | 0.893 | 0.925 | 0.952 |
| **Fold 3** | 0.910 | 0.927 | 0.894 | 0.911 | 0.948 |  | 0.816 | 0.924 | 0.808 | 0.418 | 0.899 |  | 0.910 | 0.927 | 0.894 | 0.911 | 0.948 |
| **Fold 4** | 0.917 | 0.964 | 0.872 | 0.920 | 0.946 |  | 0.790 | 0.949 | 0.777 | 0.392 | 0.886 |  | 0.917 | 0.964 | 0.872 | 0.920 | 0.946 |
| **Fold 5** | 0.913 | 0.954 | 0.872 | 0.915 | 0.946 |  | 0.800 | 0.932 | 0.790 | 0.400 | 0.882 |  | 0.913 | 0.954 | 0.872 | 0.915 | 0.946 |
| **NB** |  |  |  |  |  |  |  |  |  |  |  |  |  |  |  |  |  |
| **Fold 1** | 0.683 | 0.745 | 0.623 | 0.670 | 0.739 |  | 0.402 | 0.839 | 0.369 | 0.167 | 0.649 |  | 0.683 | 0.745 | 0.623 | 0.670 | 0.739 |
| **Fold 2** | 0.677 | 0.670 | 0.684 | 0.673 | 0.735 |  | 0.539 | 0.856 | 0.516 | 0.210 | 0.735 |  | 0.677 | 0.670 | 0.684 | 0.673 | 0.735 |
| **Fold 3** | 0.680 | 0.763 | 0.599 | 0.703 | 0.740 |  | 0.395 | 0.839 | 0.361 | 0.165 | 0.699 |  | 0.68 | 0.763 | 0.599 | 0.703 | 0.740 |
| **Fold 4** | 0.678 | 0.711 | 0.647 | 0.687 | 0.739 |  | 0.482 | 0.714 | 0.457 | 0.193 | 0.718 |  | 0.678 | 0.711 | 0.647 | 0.687 | 0.739 |
| **Fold 5** | 0.679 | 0.724 | 0.634 | 0.691 | 0.739 |  | 0.416 | 0.864 | 0.381 | 0.174 | 0.690 |  | 0.679 | 0.724 | 0.634 | 0.691 | 0.739 |
| **ANNs** |  |  |  |  |  |  |  |  |  |  |  |  |  |  |  |  |  |
| **Fold 1** | 0.707 | 0.678 | 0.736 | 0.697 | 0.771 |  | 0.763 | 0.551 | 0.781 | 0.250 | 0.720 |  | 0.707 | 0.678 | 0.736 | 0.697 | 0.771 |
| **Fold 2** | 0.710 | 0.707 | 0.713 | 0.707 | 0.763 |  | 0.745 | 0.712 | 0.748 | 0.285 | 0.806 |  | 0.71 | 0.707 | 0.713 | 0.707 | 0.763 |
| **Fold 3** | 0.711 | 0.717 | 0.706 | 0.711 | 0.773 |  | 0.753 | 0.585 | 0.766 | 0.252 | 0.722 |  | 0.711 | 0.717 | 0.706 | 0.711 | 0.773 |
| **Fold 4** | 0.705 | 0.692 | 0.718 | 0.699 | 0.768 |  | 0.744 | 0.627 | 0.753 | 0.259 | 0.751 |  | 0.705 | 0.692 | 0.718 | 0.699 | 0.768 |
| **Fold 5** | 0.703 | 0.770 | 0.637 | 0.720 | 0.769 |  | 0.678 | 0.652 | 0.681 | 0.224 | 0.708 |  | 0.703 | 0.770 | 0.637 | 0.720 | 0.769 |

LR, Logistic regression; RF, Random Forest; SVM, Support Vector Machine; XGB, eXtreme Gradient Boosting; NB, Naive Bayes; DT, decision trees; ANNs, artificial neural networks; AUC, the area under the curve under the characteristics of the subjects.

**Table S12. The DeLong test compares results across the models.**

| **Method** | **LR** | **RF** | **SVM** | **XGB** | **DT** | **NB** | **ANNs** |
| --- | --- | --- | --- | --- | --- | --- | --- |
| **LR** | 1 | **2.2e-16** | **0.005159** | **2.2e-16** | **1.236e-14** | 0.1986 | 0.4283 |
| **RF** | **2.2e-16** | 1 | **4.461e-06** | **0.0004375** | **0.01819** | **2.2e-16** | **3.343e-10** |
| **SVM** | **0.005159** | **4.461e-06** | 1 | **3.876e-08** | **0.003243** | **4.212e-05** | 0.0838 |
| **XGB** | **2.2e-16** | **0.0004375** | **3.876e-08** | 1 | **5.717e-06** | **4.212e-05** | **1.996e-13** |
| **DT** | **1.236e-14** | **0.01819** | **0.003243** | 5.717e-06 | 1 | **2.619e-12** | **1.395e-06** |
| **NB** | 0.1986 | **2.2e-16** | **4.212e-05** | **4.212e-05** | **2.619e-12** | 1 | 0.08782 |
| **ANNs** | 0.4283 | **3.343e-10** | 0.0838 | **1.996e-13** | **1.395e-06** | 0.08782 | 1 |

*Note. LR, Logistic regression; RF, Random Forest; SVM, Support Vector Machine; XGBoost, eXtreme Gradient Boosting; NB, Naive Bayes; DT, decision trees; ANNs, artificial neural networks;

**Table S13. Runtimes of all models.**

| **Model** | **XGBoost** | **RF** | **SVM** | **LR** | **DT** | **NB** | **ANNs** |
| --- | --- | --- | --- | --- | --- | --- | --- |
| **Runtime(s)** | 1.56 | 6.61 | 7.91 | 0.02 | 0.16 | 0.00 | 234 |

LR, Logistic regression; RF, Random Forest; SVM, Support Vector Machine; XGB, eXtreme Gradient Boosting; NB, Naive Bayes; DT, decision trees; ANNs, artificial neural networks.
